# Preliminary efficacy, feasibility and safety of intra-umbilical oxytocin to reduce the time to placental delivery at caesarean section: an exploratory randomized trial

**DOI:** 10.4314/ahs.v23i3.3

**Published:** 2023-09

**Authors:** Katrin Middleton, Fungai Mbengo, Thandisizwe Redford Mavundla, George Justus Hofmeyr

**Affiliations:** 1 Department of Obstetrics and Gynaecology, Walter Sisulu University, South Africa; 2 School of Nursing and Midwifery, Edith Cowan University, Western Australia, Australia; 3 Department of Nursing Education, Faculty of Health Sciences, University of the Witwatersrand, South Africa; 4 University of Botswana, Effective Care Research Unit, University of the Witwatersrand, University of Fort Hare, Walter Sisulu University, and Eastern Cape Department of Health, South Africa

**Keywords:** Caesarean section, intra-umbilical oxytocin, postpartum haemorrhage

## Abstract

**Background:**

Delayed placental separation either after vaginal birth or caesarean birth is an important cause of postpartum haemorrhage, among other causes such as uterine atony. Intra-umbilical oxytocin has been shown to reduce the time to placental delivery after vaginal birth. However, the efficacy of intra-umbilical oxytocin to reduce the time to placental delivery following caesarean section birth is not known.

**Objectives:**

To explore the preliminary efficacy, feasibility and safety of intra-umbilical oxytocin to reduce the time to placental delivery at caesarean section.

**Methods:**

A double-blind, placebo-controlled, exploratory randomized clinical trial was conducted at a tertiary hospital in the Eastern Cape Province, South Africa. A total of 66 women undergoing elective caesarean section were enrolled in the study and randomized into oxytocin group (n = 33) receiving an intra-umbilical infusion of 20 units of oxytocin in 30ml saline, and placebo group (n = 33) receiving an intra-umbilical infusion of 30ml saline. Data were analysed using Epi Info and RevMan software. Preliminary efficacy was assessed by examining the time elapsed from birth of the baby to complete delivery of the placenta; blood loss more than 500 ml; the need for manual removal of the placenta; and the completeness of the placenta. Feasibility was determined by observing the successful insertion of the catheter and injection of the solution. Safety was evaluated by investigating adverse effects of the procedure.

**Results:**

Four women (12%) in the placebo group had a delayed placental delivery compared to one (3%) in the oxytocin group. The mean time from birth to placental delivery was 159 (SD 61) seconds in the placebo group and 143 (SD 45) seconds in the oxytocin group. There was no statistically significant difference between the two groups. Feasibility of the procedure was confirmed by successful insertion of the catheter and injection of the majority of the solution in all 66 cases. No adverse effects of the procedure were identified.

**Conclusion:**

Administration of intra-umbilical oxytocin is feasible, safe and has potential to reduce the time of placental delivery at caesarean section. Further studies involving larger sample sizes are justified.

## Introduction

Postpartum haemorrhage is the primary cause of nearly one quarter of all maternal deaths worldwide and the leading cause of maternal mortality in low-income countries.^1^,^2^ In South Africa, postpartum haemorrhage associated with caesarean section is the only cause of maternal mortality that is on the increase.^3^ The South African Saving Mothers 2014-2016 report revealed that maternal deaths due to bleeding at caesarean section represent 30 per cent of all women who die due to obstetric haemorrhage.^4^

Delayed placental separation is an important cause of postpartum haemorrhage following caesarean birth, 5,6 among other causes such as uterine atony. Appropriate management of retained placenta can prevent the occurrence of postpartum haemorrhage. Methods of managing retained placenta at caesarean section include continuous cord traction, manual removal and placental drainage.^7^ Continuous traction on the umbilical cord is the preferred method to deliver the placenta at caesarean section as it is associated with less blood loss, less endometritis and shorter hospital stay than manual removal.^7^ However, cord traction takes time, which adds to the total duration of the operation. Administration of intra-umbilical oxytocin may reduce the time to placental delivery at caesarean section by transplacental delivery of oxytocin directly to the placental bed myometrium. Another advantage would be the avoidance of the routine intravenous bolus injection of oxytocin at caesarean section which itself can contribute to maternal morbidity.

Intra-umbilical vein injection for the treatment of a retained placenta was first described in 1826 by Mojon and Asdrubali.^8^ Several small and large trials were conducted to investigate this technique and its effectiveness in the management of third stage of labour.^9^-^11^ Most trials concentrated on the administration of intra-umbilical oxytocin for the management of a retained placenta rather than for the routine administration in the third stage of labour.^9^-^11^ A 2011 Cochrane systematic review found that injection of intra-umbilical oxytocin into the umbilical vein could reduce the need for manual removal of retained placenta after vaginal birth.^12^ However, restriction of the analysis to high-quality randomized trials showed that the use of oxytocin has little or no effect.^12^ A subsequent trial could not confirm the beneficial effect of intra-umbilical oxytocin.^9^ More recently, intra-umbilical carbetocin 100mcg in 20ml normal saline was found to be more effective than oxytocin 20IU in 20ml saline for management of retained placenta.^10^ One trial found intra-umbilical oxytocin in 50ml saline for routine management of third stage of labour to be more effective than intra-umbilical saline alone, and the effect was dose-related from 10IU to 30IU.^11^ To our knowledge no trials have been reported on the use of intra-umbilical oxytocin at caesarean section.

The purpose of this study was to explore the preliminary efficacy, feasibility and safety of intra-umbilical oxytocin to reduce the time to placenta delivery at caesarean section with a view to informing the need for a larger trial.

## Methods

### Study design and setting

The study used a double-blind, placebo-controlled, exploratory randomized clinical trial design and was conducted at a tertiary hospital in the Eastern Cape Province, South Africa. The hospital is a major referral healthcare facility in the province with approximately 6000 deliveries per annum.

### Participants

Participants included women undergoing elective caesarean section at the hospital between November 2009 and February 2010. Eligibility criteria included pregnancy greater than 34 weeks gestation and a live fetus. Excluded were women with placental abruption, placenta praevia, placenta accreta/increta/percreta, vasa praevia, high risk of postpartum haemorrhage, severe maternal medical condition, known life-threatening fetal abnormalities, evidence of fetal distress, evidence of labour, and women who declined to participate or were unable to give consent. Overall, a total of 66 women were enrolled in the study.

### Procedure

The study was approved by the Walter Sisulu University Faculty of Health Sciences Postgraduate Education, Training, Research and Ethics Committee, reference number: 076/09. Written permission was obtained from the hospital's management. Informed consent was obtained from all the women who participated in the study.

**Figure 1:**
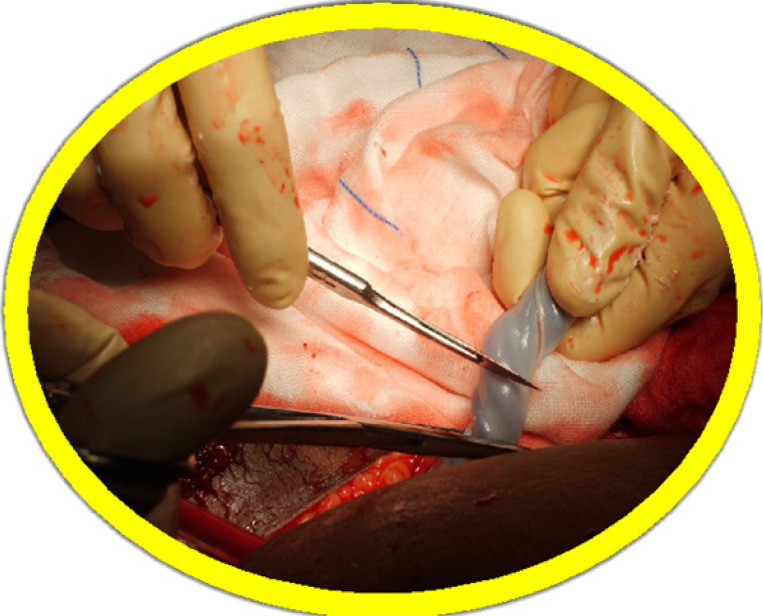
Transverse incision of the umbilical vein

**Figure 2:**
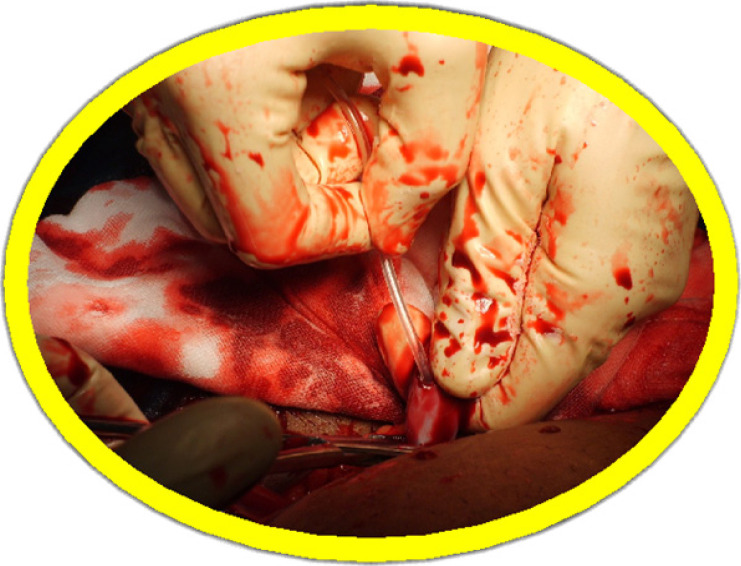
Insertion of the infant feeding tube into the umbilical vein

The eligible women were randomly allocated to two groups: the oxytocin group (n = 33) received an intra-umbilical infusion of 20 units of oxytocin in 10ml saline followed by 20ml saline; and the placebo group (n = 33) received an intra-umbilical infusion of 30ml saline. A computer-generated random sequence was used by an independent researcher to prepare a numbered series of indistinguishable ampoules with oxytocin 20 units, or saline. The trial solution was held in a secure refrigerator in the labour ward, near to the caesarean section theatre. All included women received a routine intravenous infusion of oxytocin 20 units added to 200ml saline at 100ml per hour after delivery of the placenta. Just before commencing the caesarean section, the surgeon entered the next ampoule number and the patient's details into the trial register. This was followed by drawing up the trial solution from the chosen ampoule and normal saline was added to make up 10 ml. This was all done under sterile conditions. Another syringe with 20 ml of plain normal saline was prepared as well. The first syringe with the trial solution was attached to an infant feeding tube size 8. The infant feeding tube was pre-filled with the trial solution. The caesarean section was conducted according to standard practice procedures. The anesthetist was asked to prepare an infusion of 20 units of oxytocin diluted in 200 ml of normal saline. Thirty seconds after delivery of the baby, the cord was clamped. The umbilical vein was partially incised transversely with a scalpel. The infant feeding tube was inserted into the umbilical vein until resistance was felt, withdrawn by 5cm and the fluid injected (figure 1 and 2). The whole content of the 10ml syringe was infused while the umbilical cord was manually compressed around the catheter to avoid any back-flow. This was followed by another infusion of 20 ml of normal saline to achieve complete capillary filling in the placental bed. The cord was then re-clamped on the placental side of the catheter insertion, and steady traction of 1-2 kg was applied until the placenta was delivered. After complete delivery of the placenta the anesthetist was asked to start the prepared oxytocin infusion at a rate of 100 ml per hour. The time from birth of the baby to complete delivery of the placenta was measured in seconds and recorded in the trial register for each individual.

### Data management and analysis

The collected data were entered on a spreadsheet using Microsoft Excel. Epi Info and RevMan software were used to analyse the data. Preliminary efficacy was evaluated by examining the time elapsed from birth of the baby to complete delivery of the placenta; blood loss more than 500 ml; the need for manual removal of the placenta; and the completeness of the placenta. Feasibility was assessed by observing the successful insertion of the catheter and injection of the solution. Safety was determined by investigating adverse effects of the procedure.

## Results

### Socio-demographic characteristics of the participants

As shown in Table 1, both groups were well balanced for socio-demographic and clinical variables at baseline. The main co-morbidity was pre-eclampsia with 11 (33%) women affected in the oxytocin group and 8 (24%) in the placebo group. The most frequent indication for caesarean section in both groups was previous caesarean section: 27 (82%) in the oxytocin group and 29 (88%) in the placebo group.

**Table 1:** Socio-demographic characteristics of the participants

Variable	Oxytocin group (n=33)	Placebo group (n=33)
Mean age (years)	29 (SD 7)	32 (SD 5)
Mean weight (kg)	80 (SD 18)	89 (SD 19)
Nulliparous women	4 (12%)	1 (3%)
1 previous caesarean section	18 (55%)	21 (64%)
>1 previous caesarean section	8 (24%)	10 (30%)
Preeclampsia	11 (33%)	8 (24%)
Indication for caesarean section: previous caesarean section	27 (82%)	29 (88%)
Hypertensive disorder of pregnancy	3 (9%)	3 (9%)
Other	3 (9%)	1 (3%)

SD – standard deviation

### Preliminary efficacy of intra-umbilical oxytocin

As indicated in Table 2, the mean time from birth to placental delivery was 159 seconds in the placebo group and 143 seconds in the oxytocin group. The difference was not statistically significant. The risk ratio [RR] for delayed placental delivery >3 minutes was 0.7 (95% confidence interval [CI] 0.3 to 1.6), and for delay >4 minutes the RR was 0.3 (95% CI 0.03 to 2.1). Blood loss of more than 500ml was less in the oxytocin group compared to the placebo group but did not reach statistical significance (RR 0.7, 95% CI 0.3 to 1.4). The need for manual removal of the placenta was low in both groups with a relative risk of 1.5 and a 95% CI of 0.3 to 8.4. In most of the cases in both groups the placenta was completely delivered with no significant difference in incomplete removal with a relative risk of 1 and a 95% CI of 0.2 to 6.7.

**Table 2:** Outcome data expressed as mean values (standard deviation) or proportions (%)

	Oxytocin group (n=33)	Placebo group (n=33)	P	Risk Ratio	95% Confidence Interval
Mean time from birth to placental delivery (sec)	143 (SD 45)	159 (SD 61)	0.2 (ANOVA)		
Delayed placental delivery (>3min)	7 (21%)	10 (30%)	0.6 (Chi Square)	0.7	0.3 to 1.6
Delayed placental delivery (>4min)	1 (3%)	4 (12%)	0.2 (Fisher's)	0.3	0.03 to 2.1
Blood loss ≥500 ml	8 (24%)	12 (36%)	0.3 (Chi Square)	0.7	0.3 to 1.4
Need for manual removal of placenta	3 (9%)	2 (6%)	0.5 (Fisher's)	1.5	0.3 to 8.4
Placenta not complete	2 (4%)	2 (4%)	1.0 (Fisher's)	1.0	0.2 to 6.7
Problem with infusion	4 (12%)	3 (9%)	0.5 (Fisher's)	1.33	0.3 to 5.5

### Feasibility and safety of intra-umbilical oxytocin

Feasibility of the procedure was confirmed by successful insertion of the catheter and injection of the majority of the solution in all 66 cases. Problems when injecting the infusion occurred in 4 cases (12%) in the oxytocin group and in 3 cases (9%) in the placebo group. This was caused by minimal spillage of the saline infusion around the incision at the umbilical cord. No adverse effects of the procedure were identified.

## Discussion

This study explored the preliminary efficacy, feasibility and safety of intra-umbilical oxytocin to reduce the time to placental delivery at caesarean section. The findings of this study suggest that the administration of intra-umbilical oxytocin is feasible, safe and has potential to reduce the time of placental delivery at caesarean section. Only 21% women in the oxytocin group had a delayed placental delivery more than 3 minutes compared to 30% in the placebo group. The blood loss of more than 500 ml in the oxytocin group was less than in the placebo group (24% versus 36%). These results are consistent with beneficial effects, but were not statistically significant.

The dose of 20 units oxytocin was chosen to ensure adequate dosage to the placental bed, assuming that transplacental transfer would be incomplete. However, a subsequent study has shown increasing efficacy of intra-umbilical oxytocin up to 30 units.^11^ The latter study also used a larger fluid volume (50ml).

A number of studies have been done to examine the effect of intra-umbilical oxytocin on retained placenta after vaginal birth.^9^-^11^ A recent trial found 66.7% (64/96) overall success rate for the removal of the retained placenta using oxytocin.^9^ The Cochrane Database of Systematic Reviews supported the use of intra-umbilical oxytocin in a saline solution for the treatment of a retained placenta.^12^ The subsequent “Release Study” could not replicate those results.^13^ whereas other trials found the administration of intra-umbilical oxytocin to be beneficial.^14^-^16^ Our study adds novel information on the potential, feasibility and safety of intra-umbilical uterotonic administration to assist placental separation at caesarean section.

Intravenous bolus injections of oxytocin can cause severe maternal hypotension due to the cardiovascular effects of oxytocin. It has been recommended that oxytocin administration at caesarean section should be by slow infusion. This will improve the safety for the mother, but may delay the time for placental separation compared to the use of a rapid bolus injection. If confirmed to be effective, intra-umbilical oxytocin could constitute an alternative for targeted delivery of oxytocin to the placental bed, particularly in compromised patients to avoid systemic blood pressure changes associated with bolus intravenous infusion.

The study has limitations which must be recognized when interpreting the findings. The sample size used in this pilot study was small, hence the power of the study to detect modest differences between the two groups was limited. A minimal spilling of the infusion solution occurred in only a few cases and was unlikely to affect the results.

Even with these limitations, this study contributes to knowledge on the administration of intra-umbilical oxytocin to expedite delivery of the placenta at caesarean section. The study is insufficient to recommend that intra-umbilical oxytocin be used routinely at caesarean section but demonstrates potential as an alternative if there is a delay in placental separation. Further research involving larger sample sizes is warranted to determine the efficacy of intra-umbilical oxytocin to facilitate placental delivery at caesarean section with greater certainty.
